# Role of Indigenous Bacteria in Corrosion of Two Types of Carbon Steel

**DOI:** 10.3390/microorganisms10122451

**Published:** 2022-12-12

**Authors:** Mihaela Marilena Stancu

**Affiliations:** Institute of Biology Bucharest of Romanian Academy, 296 Splaiul Independentei, P.O. Box 56-53, 060031 Bucharest, Romania; mihaela.stancu@ibiol.ro

**Keywords:** bacteria, carbon steel, biocorrosion, biofilms, rust, pitting

## Abstract

This study aimed to investigate the presence of both aerobic and anaerobic bacteria in a water sample collected from a nuclear power plant and establish if the indigenous bacteria or the products of their metabolic activities could initiate the corrosion of two different types of carbon steel (i.e., A570, 1045). The aerobic (heterotrophic, iron-oxidizing) and anaerobic (sulfate-reducing) bacteria were detected in low numbers in the water sample. Three bacterial strains were isolated by the enrichment procedure from this sample. Based on phenotypic and genotypic characteristics, the isolated bacteria were identified as *Stenotrophomonas maltophilia* IBB_Cn1_ (MT893712), *Stenotrophomonas maltophilia* IBB_Cn2_ (MT893713), and *Bacillus thuringiensis* IBB_Cn3_ (MT893714). The bacteria existing in the water sample were able to initiate the corrosion of carbon steel A570 and 1045. The sulfate-reducing bacteria were detected in higher numbers than the heterotrophic bacteria and iron-oxidizing bacteria at the end of the biocorrosion experiments. The carbon steel coupons revealed macroscopic and microscopic changes in the surface characteristics, and these changes could be due to biofilm formation on their surfaces and the accumulation of the corrosion products. The corrosion rate varied from one type of carbon steel to another, depending on the incubation conditions and the chemical composition of the coupons.

## 1. Introduction

In natural and human-made environments, metal and alloy corrosion ensue due to chemical or electrochemical interactions between the metal and its environment [1,2,3]. Microbially influenced corrosion (MIC), also known as biocorrosion, is a type of corrosion in which the deterioration of metal is initiated by the presence of microorganisms such as bacteria and the products of their metabolic activities [4,5,6,7,8]. Many bacteria tend to attach to metal surfaces and form biofilms, which create conditions that can initiate and accelerate metal corrosion and cause severe damage to the metal [9,10,11]. Additionally, inorganic precipitates resulting from the bulk aqueous phase and corrosion products are also present in biofilms. Biofilms have exceptional abilities to adapt and survive under extreme values of temperature, salinity, pH, redox potential, pressure, and radiation [7,12]. Bacteria and the products of their metabolic activities (e.g., exopolymers, enzymes, organic and/or inorganic acids, volatile compounds) can affect the anodic and cathodic reactions at metal surfaces, thus altering the electrochemical processes at the biofilm and metal interface [7,8,10,13]. 

Carbon steel is an iron (Fe) and carbon (C) alloy that contains about 98% Fe, less than 2% C, and other elements in small quantities, such as silicon (Si), phosphorus (P), sulfur (S), manganese (Mn), aluminum (Al), etc. [1]. The alloying elements confer the required properties, such as tensile strength, hardness, or corrosion resistance. Due to its tensile strength and low cost, carbon steel is a significant component used in many industries, including in several energy industries, such as the fossil fuel and nuclear power industries (in reactor pressure vessels, steam generator shells, turbine casings, etc.) [3,6]. Although biocorrosion has been known for many years, it has been accepted as a critical corrosion mechanism for nuclear power plants for only about 30 years. The design characteristics of reactors with redundant static water systems, together with long shut-down periods, make nuclear power plants susceptible to biocorrosion [14]. Both aerobic (e.g., slime-, exopolymers- or acid-producing bacteria, iron-oxidizing bacteria, manganese-oxidizing bacteria) and anaerobic bacteria (e.g., sulfate-reducing bacteria, iron-reducing bacteria) can cause localized corrosion on carbon steel surfaces through the formation of biofilms [4,5,6,7,9,11,15]. The presence of sulfate-reducing bacteria, iron-oxidizing bacteria, and aerobic heterotrophic bacteria in the cooling circuit of a nuclear reactor and how these bacteria were involved in the carbon steel corrosion was previously reported by Rao et al. [16]. From these three groups, sulfate-reducing bacteria are the main bacteria responsible for carbon steel corrosion, and their presence is often considered a marker for biocorrosion in cooling water systems [12].

Nuclear reactors are complex systems of different connected materials that need to work optimally to ensure the efficient and safe operation of nuclear power plants. Even under careful nuclear power plant operation, the corrosion of the plant components is inevitable, and the metals tend to turn back to their thermodynamically stable state, such as hematite (Fe_2_O_3_), which is the basis of the appearance of rust on metal surfaces [14]. The biocorrosion of carbon steel is a serious issue in many countries due to enormous economic damage and costs associated with the repair and replacement of the affected metallic materials. While the bacteria responsible for MIC usually exist in the environment in synergistic communities of different species, most laboratory-based MIC experiments have been performed with single bacterial pure cultures [8]. This study aimed to investigate the presence of both aerobic and anaerobic bacteria in a water sample collected from a nuclear power plant and to establish if the indigenous bacteria or the products of their metabolic activities could initiate the corrosion of two different types of carbon steel, A570 and 1045 (ASTM-AISI). The first type is general-purpose carbon steel, and the second type is medium carbon steel, usually used in areas requiring greater strength and hardness. To the best of the author’s knowledge, this is the first report to investigate the role of indigenous aerobic and anaerobic bacteria in the corrosion of two different types of carbon steel.

## 2. Materials and Methods

### 2.1. Bacteria Quantification in the Water Sample

The non-radioactive water sample used in this study was collected from the Cernavodă Nuclear Power Plant (distribution basin, Constanța County, Romania). The pH of the water sample was measured using a Hanna bench pH 213 (Woonsocket, RI, USA). The number of bacteria in the water sample was determined through a modified most probable number (MPN) assay described by Stancu and Grifoll [17]. Briefly, the 96-well microtiter plates containing 180 µL liquid nutrient-rich (i.e., LB) and nutrient-poor (i.e., Winogradsky, Postgate) culture medium and 20 µL aliquots of 10-fold dilutions of the sample were incubated for 3–10 days at 30 °C, under aerobic or anaerobic conditions. Here, as elsewhere in this study, the anaerobic conditions were created by covering the surface of the culture medium (i.e., Postgate) with sterile paraffin oil. The growth of bacteria (cells mL^−1^) was determined using triphenyl tetrazolium chloride (TTC) dye as a redox indicator of cell respiration [18]. to Each well was added with 0.3% TTC and then the plates were incubated for one more day, in the dark, at room temperature. The wells were considered positive for growth when a red color developed as a result of TTC reduction. Here, as elsewhere in this study, the assays were done in duplicate.

Luria–Bertani (LB) culture medium [19] containing 10.0 g tryptone, 5.0 g yeast extract, and 10.0 g NaCl per liter of distilled water was used to quantify heterotrophic bacteria (HB). Winogradsky medium [20] containing 0.5 g NH_4_NO_3_, 0.5 g NaNO_3,_ 0.5 g K_2_HPO_4_, 0.5 g MgSO_4_·7H_2_O, 0.2 g CaCl_2_·6H_2_O, and 10.0 g ferric ammonium citrate per liter of distilled water was used to quantify iron-oxidizing bacteria (IOB). Postgate medium [21] containing 0.5 g KH_2_PO_4_, 1.0 g NH_4_Cl, 1.0 g CaSO_4_·2H_2_O, 2.0 g MgSO_4_·7H_2_O, 3.5 g calcium lactate, 1.0 g yeast extract, 0.5 g FeSO_4_·7H_2_O, and 0.02 g Na_2_S·9H_2_O per liter of tap water was used to quantify sulfate-reducing bacteria (SRB). For the solid medium, 20.0 g agar was added per liter of liquid medium. Reagents used in this study were purchased from Merck (Darmstadt, Germany), and Sigma-Aldrich (Saint-Quentin-Fallavier, France).

### 2.2. Isolation and Identification of Bacteria from the Water Sample 

#### 2.2.1. Isolation of Bacteria

The bacteria were isolated from the water sample through the enrichment procedure in nutrient-rich (i.e., LB) and nutrient-poor (i.e., Winogradsky, Postgate) culture medium. The tubes containing 20 mL liquid culture medium and 2 mL of the sample were incubated for 3–10 days at 30 °C under aerobic or anaerobic conditions. Then, 100 µL aliquots of 10-fold dilutions of the enrichment cultures were spread on the corresponding solid medium (i.e., LB, Winogradsky, Postgate). The bacteria were purified by repeatedly streaking on the corresponding solid medium and stored frozen in 25% glycerol at −80 °C.

#### 2.2.2. Identification of Bacteria

The newly isolated bacteria were identified through their phenotypic (i.e., the color of the colonies, Gram, shape, motility, respiratory type, catalase, oxidase, hydrogen sulfide production, pyocyanin and pyoverdin pigment production, growth on TTC, lactose utilization [22]) and genotypic characteristics (i.e., genomic fingerprinting, 16S rRNA gene sequencing). For genotypic characterization, the genomic DNA was extracted from the isolated bacteria using the Pure Link genomic kit (Invitrogen, Carlsbad, CA, USA).

The random amplification of DNA fragments (RAPD) was carried out using genomic DNA, AP5 [23], or AP12 [24] specific primers, dNTPs, and GoTaq G2 DNA polymerase (Promega, Madison, WI, USA), as described by Stancu [18]. The amplification reactions (one cycle of 94 °C, 10 min; 45 cycles of 94 °C, 1 min, 36 °C, 1 min, and 72 °C, 2 min; one final cycle of 72 °C, 10 min) were performed in a Mastercycler Pro S (Eppendorf, Hamburg, Germany). The reaction products were analyzed on 2% agarose gel [19] stained with SYBR Safe (Invitrogen, Carlsbad, CA, USA).

The polymerase chain reaction (PCR) amplification of the 16S rRNA gene from the genomic DNA was carried out using 27f and 1492r universal bacterial primers [25], dNTPs, and GoTaq G2 DNA polymerase (Promega), as described by Stancu [18]. The amplification reactions (one cycle of 94 °C, 10 min; 35 cycles of 94 °C, 1 min, 55 °C, 30 s, and 72 °C, 2 min; one final cycle of 72 °C, 10 min) were performed in a Mastercycler Pro S (Eppendorf, Hamburg, Germany). The reaction products were analyzed on 1.5% agarose gel [19] stained with SYBR Safe (Invitrogen, Carlsbad, CA, USA). The reaction products were gel-purified and subsequently subjected to sequencing (CeMIA SA, Larissa, Greece). The 16S rRNA gene sequences of the newly isolated bacteria were analyzed using the BLASTN program to identify the most similar sequence available in the NCBI database [26] and then deposited in GenBank under the accession numbers MT893712, MT893713, and MT893714.

### 2.3. Biocorrosion of Carbon Steel Coupons Exposed to the Water Sample

Two types of rectangular carbon steel coupons, A570 (*c1*, *c2*) and 1045 (*c3*, *c4*), were used in this study. The coupons (with an average area of 4.06 cm^2^) were primarily sterilized by immersion in 70% ethanol (15 min), degreased in 100% ethanol, air-dried, weighed, and sterilized under ultraviolet light (15 min on each side) in the sterile air stream [27]. Then, the coupons were immersed in the water sample. The tubes were incubated for 120 days at 30 °C under aerobic or anaerobic conditions. The anaerobic conditions were created by covering the surface of the water with sterile paraffin oil. Abiotic controls were prepared similarly but using filter-sterilized water (0.22 μm pore filter).

Carbon steel A570 contains 99.29% Fe, 0.15% C, and 0.02–0.40% other elements (i.e., 0.09% Si, 0.40% Mn, 0.02% P, 0.02% S, 0.02 Al), while carbon steel 1045 contains 98.32% Fe, 0.48% C, and 0.01–0.79% other elements (i.e., 0.03% Si, 0.79% Mn, 0.02% P, 0.03% S, 0.03 Al, 0.05% Ni, 0.06% Cr, 0.18% Cu, 0.01% Sn, 0.01% As) [28].

#### 2.3.1. Bacteria Quantification during the Corrosion Experiments

The number of bacteria in the samples was studied by MPN assay as described above. The growth of the bacteria was also studied by spot assay [17]. Petri plates containing 20 mL solid culture medium (i.e., LB, Winogradsky, Postgate) were spot-inoculated with 20 µL of the samples, air-dried, and incubated for 1–3 days at 30 °C, under aerobic or anaerobic conditions. The growth of the bacteria was expressed as colony formation on a solid medium.

The pH of the samples was measured using a Hanna bench pH 213. The samples were centrifuged (20 min), and then the pH of the supernatant of each sample was measured.

#### 2.3.2. X-ray Fluorescence (XRF) Spectrometry

During the biocorrosion experiments, the quantification of the elements in the samples was carried out using a Rigaku benchtop WDXRF (wavelength dispersive X-ray fluorescence) spectrometer (Rigaku Corp., Tokyo, Japan). The samples were centrifuged (20 min) and then the supernatant of each sample was analyzed by WDXRF.

#### 2.3.3. Isolation of Bacterial Consortia

At the end of the biocorrosion experiments, the bacterial consortia were isolated from the samples through the enrichment procedure. The tubes containing 20 mL liquid culture medium (i.e., LB) and 100 µL of the samples were incubated for 3 days at 30 °C under aerobic or anaerobic conditions. Bacterial consortia were stored frozen in 25% glycerol at −80 °C. The genomic DNA was extracted from the isolated bacterial consortia using the Pure Link genomic kit (Invitrogen, Carlsbad, CA, USA).

The RAPD was carried out using genomic DNA, AP5 [23] or AP12 [24] specific primers, dNTPs, and GoTaq G2 DNA polymerase (Promega, Madison, WI, USA) as described above. 

The PCR amplification of the 16S rRNA gene from the genomic DNA was carried out using 27f and 1492r universal bacterial primers [25], dNTPs, and GoTaq G2 DNA polymerase (Promega, Madison, WI, USA), as described above. 

#### 2.3.4. Optical Microscopy (OM), Scanning Electron Microscopy (SEM)

The carbon steel coupons were fixed in a 2% glutaraldehyde solution (60 min), washed, dehydrated in a graded series of 20–100% ethanol solution (15 min each), and air-dried [1,27]. The surface changes in the carbon steel coupons were investigated using a Zeiss Axiostar Plus 426126 optical microscope (Zeiss, Göttingen, Germany) with a 10× objective. Gold-coated coupons were also examined using a JEOL JSM-6610LV scanning electron microscope (JEOL, Peabody, MA, USA) operating at 10–20 kV.

#### 2.3.5. Weight Loss, Corrosion Rate

The weight loss of the carbon steel coupons was determined, as described by Valencia-Cantero and Peña-Cabriales [27]. The coupons were sonicated in a 5% citric acid solution (5 min) using an ultrasonic homogenizer Sonopuls GM3100 (Bandelin, Berlin, Germany) to remove the biofilm and mineral film, washed in distilled water, air-dried, and weighted. The corrosion rate was measured as the weight loss divided by the coupon area, time, and density using the equation indicated by de Melo et al. [1].

## 3. Results and Discussion

### 3.1. Bacteria Quantification in the Water Sample

**Figure 1:**
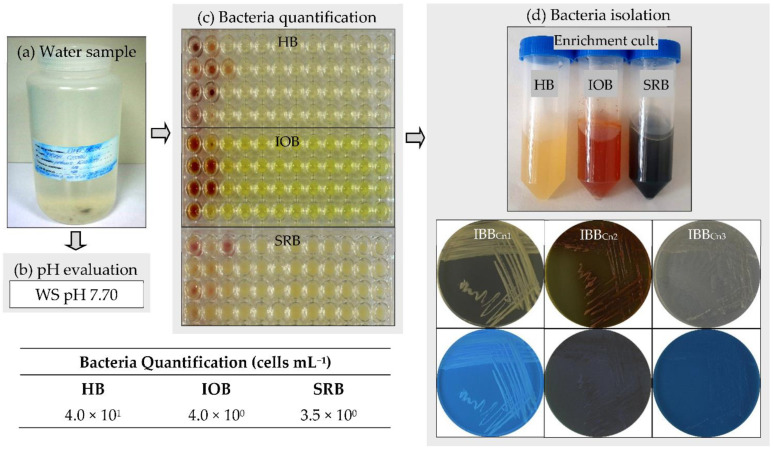
Bacteria quantification in the water sample and bacteria isolation. (**a**) Water sample. (**b**) pH evaluation of water sample (WS). (**c**) Bacteria quantification (MPN assay, cells mL^−1^): heterotrophic bacteria (HB), iron-oxidizing bacteria (IOB), sulfate-reducing bacteria (SRB); the values represent the average from two independent assays. (**d**) Bacteria isolation by the enrichment assay and their purification: *S. maltophilia* IBB_Cn1_, *S. maltophilia* IBB_Cn2_, *B. thuringiensis* IBB_Cn3_.

The water sample used in this study was collected from the Cernavodă Nuclear Power Plant, Romania, which provides about 20% of the country’s electricity. The pH of the environment is a significant parameter for the growth of bacteria. The first parameter measured was the pH of the water sample (Figure 1a,b). The sample had a slightly alkaline pH (around 7.70). Most bacteria grow in media with pH values between 6.0 and 7.0. Therefore, three different culture media, including LB (with a pH value of 7.0), Winogradsky (pH 6.0), and Postgate (pH 7.0), were used to quantify the heterotrophic bacteria (HB), the iron-oxidizing bacteria (IOB), and the sulfate-reducing bacteria (SRB), respectively. The nutrient-rich culture medium (i.e., LB) was used to quantify the presence of fast-growing bacteria (i.e., HB) in the water sample. In contrast, nutrient-poor culture media (i.e., Winogradsky, Postgate) were used to quantify the presence of slow-growing bacteria (i.e., IOB, SRB). Since the traditional solid-based culture methods have their limitations [29], a liquid-based culture method (i.e., 96-well microtiter plates MPN assay) was used for bacteria quantification in the water sample collected from the Cernavodă Nuclear Power Plant (Romania). Many bacteria are well known to be nutrient-specific and require culture media with well-defined chemical composition for optimal growth. It is well-recognized that only about 1% of bacteria from various environments can be cultivated in vitro [30]. In the water sample, the most probable number per mL was very low (below 4.0 × 10^1^ cells mL^−1^) for all the tested bacteria (Figure 1c). However, a higher number was acquired for heterotrophic bacteria (4.0 × 10^1^ cells mL^−1^) than those for iron-oxidizing (4.0 × 10^0^ cells mL^−1^) and sulfate-reducing bacteria (3.5 × 10^0^ cells mL^−1^). The bacteria are the most important microorganisms involved in metal corrosion in several aqueous systems [4,7,8,9,15]. Furthermore, the presence of both aerobic (e.g., exopolymers-producing, iron-oxidizing) and anaerobic (e.g., sulfate-reducing) bacteria in a water sample leads to corrosion rates higher than those found with a single type of bacteria [13].

### 3.2. Isolation and Identification of Bacteria from the Water Sample

**Figure 2:**
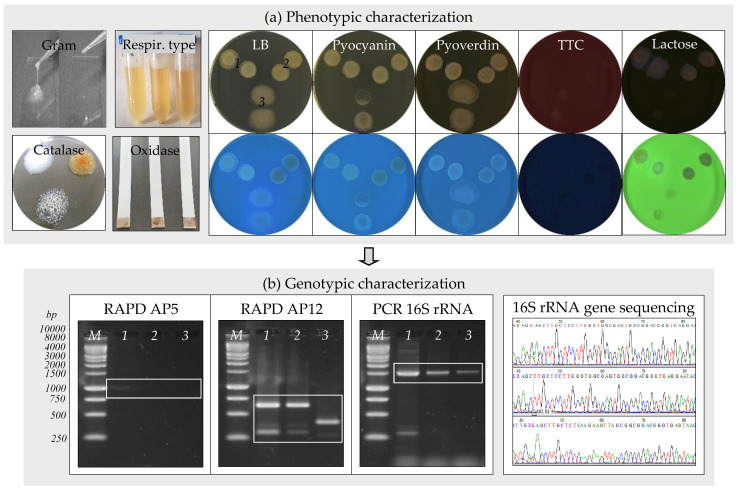
Bacteria identification through phenotypic and genotypic characteristics. (**a**) Phenotypic characterization: *S. maltophilia* IBB_Cn1_ (*1*); *S. maltophilia* IBB_Cn2_ (*2*); *B. thuringiensis* IBB_Cn3_ (*3*). (**b**) Genotypic characterization: *S. maltophilia* IBB_Cn1_ (*1*); *S. maltophilia* IBB_Cn2_ (*2*); *B. thuringiensis* IBB_Cn3_ (*3*); RAPD using AP5 (*1*–*3*) or AP12 (*1*–*3*) primers; PCR of 16S rRNA gene (1465 bp fragment) using 27f-1492r primers (*1*–*3*); 1 kb DNA ladder, Promega (*M*).

Three bacterial strains, assigned as IBB_Cn1_, IBB_Cn2_, and IBB_Cn3_, were isolated from the water sample after enrichment in nutrient-rich and nutrient-poor culture media (Figure 1d). Strain IBB_Cn1_ was isolated by enrichment in LB medium, while the strains IBB_Cn2_ and IBB_Cn3_ were isolated by enrichment in Winogradsky and Postgate media, respectively. The phenotypic and genotypic characteristics of the isolated bacteria were further studied (Table 1, Figure 2a,b). Strain IBB_Cn1_ formed creamy-pigmented colonies on the LB medium, and it was facultative aerobic, motile Gram-negative rod-shaped, catalase, hydrogen sulfide and lactose positive, and oxidase negative. Strain IBB_Cn2_ formed red-yellow pigmented colonies on the Winogradsky medium, and it was facultative aerobic, motile Gram-negative rod-shaped, catalase and lactose positive, and hydrogen sulfide and oxidase negative. Strain IBB_Cn3_ formed white-pigmented colonies on the Postgate medium, and it was anaerobic, motile Gram-positive rod-shaped, catalase and hydrogen sulfide positive, oxidase, and lactose negative. None of the isolated bacteria could produce pyocyanin and/or pyoverdin pigments and did not grow on the TTC medium. Based on the phenotypic characteristics, the isolated bacteria were classified within the genera *Stenotrophomonas* and *Bacillus*. Because different RAPD-DNA fingerprints (size range, 250 to 1000 bp) were obtained for the IBB_Cn1_, IBB_Cn2_, and IBB_Cn3_ strains, all of them were further identified based on 16S rRNA gene sequences. The expected fragment (size 1465 bp) for the 16S rRNA gene was detected in all the DNA samples; the amplification of other unspecific fragments (i.e., size 300, 4000 bp) was also observed. Comparative analyses using the BLASTN program revealed that sequences of IBB_Cn1_, IBB_Cn2_, and IBB_Cn3_ strains had 99.78–99.85% sequences similar to *Stenotrophomonas* sp. SB341 (KJ191387.1), *Stenotrophomonas maltophilia* cqsm_h3 (MN826555.1), and *Bacillus thuringiensis* serovar. *thuringiensis* IS5056 (CP004123.1), respectively. Based on the phenotypic and genotypic characteristics, the isolated bacteria were identified as *Stenotrophomonas maltophilia* IBB_Cn1_ (MT893712), *Stenotrophomonas maltophilia* IBB_Cn2_ (MT893713), and *Bacillus thuringiensis* IBB_Cn3_ (MT893714). It was not surprising to isolate such bacteria from the studied water sample since these bacteria are ubiquitous in a wide variety of aquatic environments as a result of their genetic plasticity. Previously, the isolation of other bacterial strains from the genera *Stenotrophomonas* [31] and *Bacillus* [32] was reported to be able to initiate and accelerate metal corrosion in several environments. The spores produced by some bacteria are extremely resistant to harsh conditions (e.g., high temperatures) and can be transported in nuclear power systems and remain dormant for years before being revived to germinate, attach to metal surfaces, and create colonies that produce corrosive metabolites [14].

### 3.3. Biocorrosion of Carbon Steel Coupons Exposed to the Water Sample

**Figure 3:**
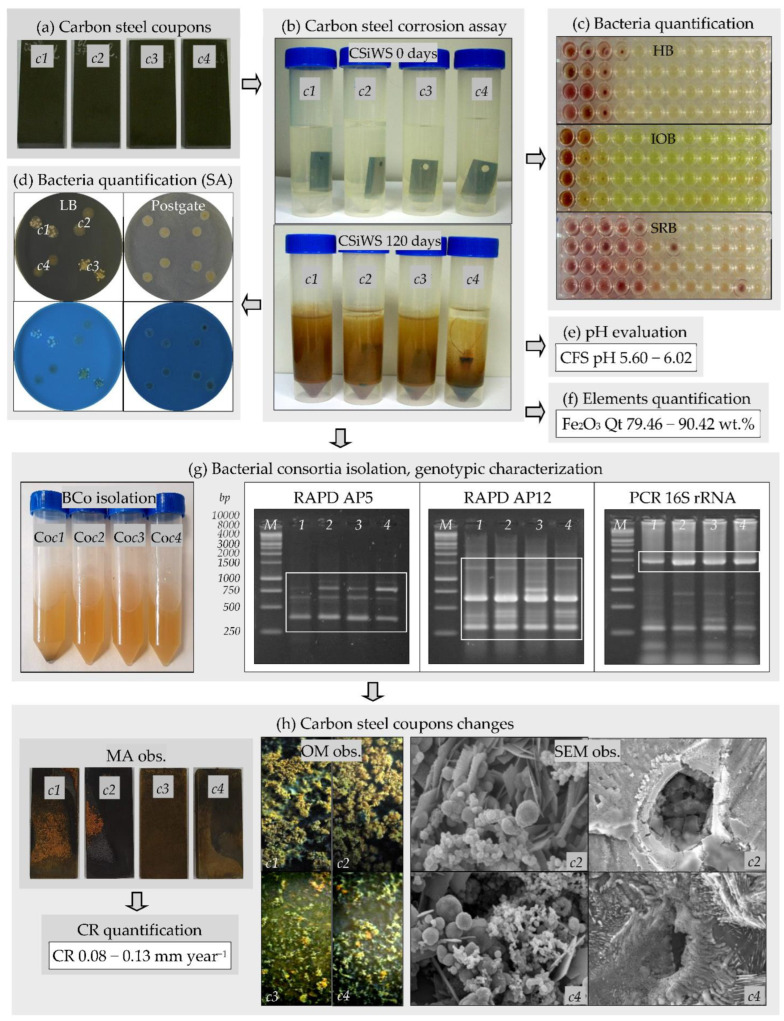
Biocorrosion of carbon steel coupons exposed to the water sample. (**a**) Carbon steel A570 (*c1*, *c2*), 1045 (*c3*, *c4*) coupons. (**b**) Carbon steel (CS) corrosion assay: CS A570 (*c1*, *c2*), 1045 (*c3*, *c4*) coupons immersed in the water sample (CSiWS) and incubated for 0−120 days under aerobic (*c1*, *c3*) or anaerobic (*c2*, *c4*) conditions. (**c**) Bacteria quantification (MPN assay): heterotrophic bacteria (HB), iron-oxidizing bacteria (IOB), sulfate-reducing bacteria (SRB). (**d**) Bacteria quantification (spot assay, SA). (**e**) pH evaluation of cell-free samples (CFS). (**f**) Element quantification (Qt) by WDXRF spectrometry, Fe_2_O_3_ Qt (wt.%). (**g**) Bacterial consortia (BCo) isolation, genotypic characterization: consortium Co*c1* (*1*); Co*c2* (*2*); Co*c3* (*3*); Co*c4* (*4*); RAPD using AP5 (*1*–*4*) or AP12 (*1*–*4*) primers; PCR of 16S rRNA gene (1465 bp fragment) using 27f-1492r primers (*1*–*4*); 1 kb DNA ladder, Promega (*M*). (**h**) Carbon steel coupon changes: macroscopic observation (MA obs.); optical microscopy observation (OM obs., magnification of ×10); scanning electron microscopy observation (SEM obs.) before (left images, ×11,000) and after (right images, ×5000) removal of biofilm and corrosion products; corrosion rate (CR) quantification of CS coupons.

The biocorrosion of carbon steel is a complex process that involves different abiotic (e.g., oxygen levels, temperature, nutrients, pH) and biotic factors through the direct and indirect action of bacteria [27]. Although the biocorrosion of carbon steel has been known for a long time, significant data gaps persist in the understanding of the fundamental mechanisms and processes leading to MIC [3,4,14]. The capacity of the bacteria that existed in the water sample to initiate the corrosion of two different types of carbon steel coupons, A570 (*c1*, *c2*) and 1045 (*c3*, *c4*), was further investigated in this study (Figure 3a–h). All the coupons were immersed for 120 days in the water sample; two of them, *c1* and *c3*, were incubated under aerobic conditions, while the other two coupons, *c2* and *c4*, were incubated under anaerobic conditions (Figure 3b). At the end of the biocorrosion experiments, in all variants, the color changes (from colorless to reddish-brown color) and the appearance of a black precipitate were observed (Figure 3b). At the beginning of the experiments, in all variants, the tested bacteria were detected at a lower number (range, 3.5 × 10^0^ to 4.0 × 10^1^ cells mL^−1^) compared to their number at the end of the experiments (range, 3.0 × 10^2^ to 5.0 × 10^5^ cells mL^−1^) (Figure 3c, Table 2). As observed, the sulfate-reducing bacteria were detected at a higher number (range, 3.0 × 10^3^ to 5.0 × 10^5^ cells mL^−1^) than the heterotrophic bacteria and iron-oxidizing bacteria (range, 3.0 × 10^2^ to 4.0 × 10^3^ cells mL^−1^) at the end of the experiments. These results were confirmed by the spot assay (Figure 3d). The high number of sulfate-reducing bacteria obtained was not unexpected. Sulfate-reducing bacteria are considered the most significant and critical corrosion-accelerating factor in the context of the biocorrosion of metals in several environments [3,8,9,11]. 

The pH of the water sample was initially 7.70. At the end of the biocorrosion experiments, lower pH values (range, 5.60 to 6.02) were obtained due to the bacteria growth under aerobic and anaerobic conditions (Figure 3e, Table 2). Thus, the bacteria from the water sample in which carbon steel A570 and 1045 coupons were immersed were able to produce corrosive metabolites, such as acids. For the abiotic controls, no changes in the pH values were observed. Several bacteria can produce organic and/or inorganic acids as end-products of their metabolism that can initiate and accelerate metal corrosion [2,4,5,6].

At the beginning of the biocorrosion experiments, the elements detected through WDXRF analyses in the water sample were Fe_2_O_3_ (5.13 wt.%), Al_2_O_3_, SiO_2_, SO_3_, Cl, K_2_O, CaO, Cr_2_O_3_, MnO, NiO, ZnO, GeO_2_, As_2_O_3_, and SeO_2_ (range, 2.48 to 19.77 wt.%). Significant differences were observed in the chemical compositions of the samples after carbon steel A570 (*c1*, *c2*) and 1045 (*c3*, *c4*) coupon immersions; the elements detected in these samples were Fe_2_O_3_ (range 79.46 to 90.42 wt.%), Al_2_O_3_, SiO_2_, SO_3_, Cl, K_2_O, CaO, Cr_2_O_3_, MnO, NiO, ZnO, GeO_2_, As_2_O_3_, SeO_2_, P_2_O_5_, V_2_O_5_, Br, Nd_2_O_3_, and Pm_2_O_3_ (range, 0.23 to 3.28 wt.%). The most significant element detected in the analyzed samples was hematite (Fe_2_O_3_), known as the best stable form of iron oxide. As observed (Figure 3f, Table 2), at the end of the biocorrosion experiments, Fe_2_O_3_ was detected in higher concentrations (79.46–90.42 wt.%), as compared to those initially detected (5.13 wt.%). Therefore, the Fe_2_O_3_ concentration was boosted about 15–18 times in the samples after the immersion of the coupons compared to the initial concentrations. For the abiotic controls, lower concentrations of Fe_2_O_3_ (38.50 wt.%) were detected compared to those detected in the biocorrosion experiments.

Four bacterial consortia, assigned as Co*c1*, Co*c2*, Co*c3*, and Co*c4*, were isolated from the water sample after the carbon steel A570 (*c1*, *c2*) and 1045 (*c3*, *c4*) coupon immersions through the enrichment in LB medium (Figure 3g). Very close RAPD-DNA fingerprints (size range, 300 to 1400 bp) were obtained for the DNA extracted from the Co*c1*, Co*c2*, Co*c3*, and Co*c4* bacterial consortia. When RAPD was performed using AP5 primer, only four distinct bands (size 400, 650, 800, 1000 bp) were obtained, while when AP12 primer was used, seven distinct bands (size 300, 400, 450, 650, 800, 1000, 1400 bp) were acquired. As compared to the RAPD-DNA fingerprints obtained for the IBB_Cn1_, IBB_Cn2_, and IBB_Cn3_ isolated strains, the Co*c1*, Co*c2*, Co*c3*, and Co*c4* consortia revealed more complex bacterial profiles, proving the presence of more than one bacterial fingerprint profile within them. The DNA extracted from these four bacterial consortia was also used as a template for the PCR amplification of the 16S rRNA gene. The expected fragment (size 1465 bp) for the 16S rRNA gene was detected in all the samples. However, the amplification of other non-specific fragments (size range, 100 to 700 bp) was also observed for all of them. 

**Figure 4:**
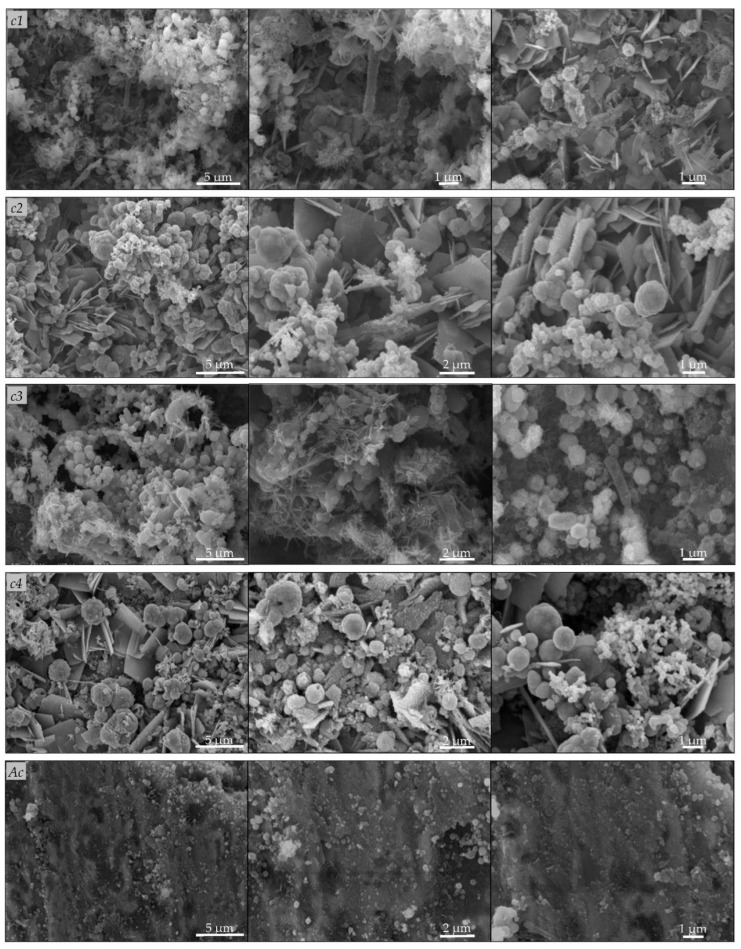
Scanning electron microscopy observation of carbon steel coupons before removal of biofilm and corrosion products. Carbon steel A570 (*c1*, *c2*), 1045 (*c3*, *c4*) coupons immersed in the water sample and incubated for 120 days under aerobic (*c1*, *c3*) or anaerobic (*c2*, *c4*) conditions; abiotic controls (*Ac*).

At the end of the biocorrosion experiments, the carbon steel coupons revealed macroscopic changes in the surface characteristics (Figure 3h), and these changes could be due to the biofilm formation on their surfaces and the accumulation of the corrosion products. Biofilms consist of bacterial cells and their extracellular polymeric substances (EPS), which are complex mixtures of cell macromolecules (e.g., polysaccharides, proteins, lipids, nucleic acids) that facilitate the irreversible attachment of cells to the metal surfaces [7]. The main forces involved in developing biofilms on metal surfaces comprise nutrient deprivation, continuous water flow, and unfavorable environmental conditions. The formation of the biofilms permits the generation of an internal microenvironment that can initiate and accelerate metal corrosion [12,33]. When the rate of oxygen diffusion in the biofilm is less than the rate of respiration, areas inside of the biofilm become anaerobic and sulfate-reducing bacteria can proliferate in the anaerobic conditions created in biofilms, such as the iron-rich tubercles produced by iron-oxidizing bacteria [34]. When the carbon steel A570 (*c1*, *c2*) coupons were immersed in the water sample, the formation of biofilms and the accumulation of the corrosion products (e.g., rust) on their surfaces were more significant compared to that observed for 1045 (*c3*, *c4*) steel (Figure 3h). The coupon changes detected by macroscopic observations were confirmed by optical microscopy and scanning electron microscopy observations (Figure 3h and Figure 4). The appearance of rust on the surfaces of the coupons immersed in the water sample was not surprising because rusting is a phenomenon that can accompany the corrosion of carbon steel. The rust layer that appears on the surfaces of carbon steel immersed in water is divided into three layers: outer (red-brown), middle (yellow), and inner (black) layers [9]. The corrosion products can be either soluble or insoluble in the aqueous solution. The essential corrosion products identified on the surfaces of other carbon steel types (e.g., AISI-1020) exposed to different aqueous systems were various forms of iron oxide hydroxides (FeOOH), magnetite (Fe_3_O_4_), and all forms of iron sulfide (Fe_x_S_y_) [1]. Goethite (α-FeOOH) and lepidocrocite (γ-FeOOH), with globular and acicular morphologies, are the main crystalline phases in the composition of rust, which are responsible for the reddish-brown color of rust [9].

**Figure 5:**
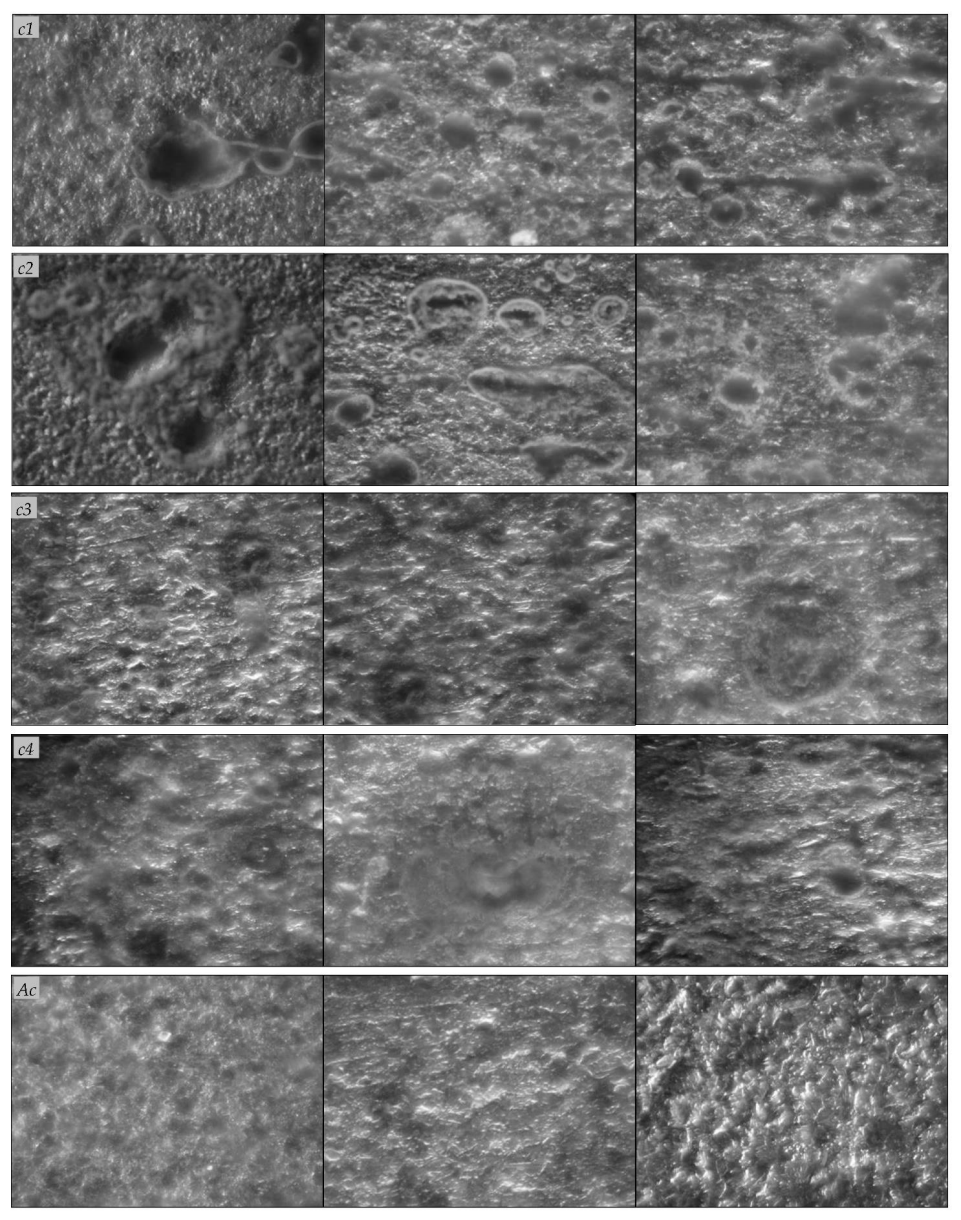
Optical microscopy observation of carbon steel coupons after the removal of biofilm and corrosion products. Carbon steel A570 (*c1*, *c2*), 1045 (*c3*, *c4*) coupons immersed in the water sample and incubated for 120 days under aerobic (*c1*, *c3*) or anaerobic (*c2*, *c4*) conditions; abiotic controls (*Ac*); magnification of ×40.

Scanning electron microscopy observation (Figure 4) of the carbon steel A570 (*c1*, *c2*) and 1045 (*c3*, *c4*) coupons revealed the formation of biofilms on the metal surfaces and the appearance of changes in the surface characteristics. On the surface of the coupons, we observed irregular heterogeneous dense mineral layers with globular, acicular, or lamellar morphologies, and other morphologies, such as stars, flowers, or rods, that are characteristic of different forms of iron oxides [9]. The corrosion products also appeared as massive, thick, and porous deposits on the carbon steel A570 (*c1*, *c2*) and 1045 (*c3*, *c4*) coupons, and the mineral layers formed on their surfaces were colonized by bacteria. On the contrary, for the abiotic controls, no such changes were observed and the corrosion products appeared as thin mineral layers on the metal surfaces (Figure 4). Previously, de Melo et al. [1] reported bacteria attachment to AISI-1020 carbon steel cylinders exposed for 15 days to different aqueous systems and localized corrosion (e.g., pits). Valencia-Cantero and Peña-Cabriales [27] reported cracks in mineral films colonized by bacteria on the surface of carbon steel 1018 coupons incubated for 25 days in the presence of bacterial consortia containing both iron-reducing and sulfate-reducing bacteria, coupled with higher corrosion rates compared to those of coupons exposed only to the sulfate-reducing consortium. On the surfaces of the carbon steel A570 (*c1*, *c2*) and 1045 (*c3*, *c4*) coupons, we observed pits and holes with different geometries after the removal of the biofilms and corrosion products, which were not observed in the abiotic controls after the removal of the corrosion products (Figure 5). Cai et al. [15] observed surface morphology changes (e.g., pits, holes) for carbon steel 1045 coupons immersed for 30 days in sterile seawater inoculated with a mixture of bacteria. Bacterial metabolites and corrosion products form a compact corrosion product film on metal surfaces, in which the transportation of corrosive species, such as oxygen, and different ions can be disturbed, and thus, exert additional influences on the corrosion rates [15].

It is well known that the corrosion rate is a very important parameter as it provides important information about the severity of the corrosion process in aquatic environments. As expected, at the end of the experiments, the corrosion rates varied from one type of carbon steel coupon to the other (A570, 1045) and even for some types of coupons depending on the incubation conditions (aerobic or anaerobic) (Table 2). Similarly, de Melo et al. [1] and Refait et al. [3] have reported that the corrosion rates varied depending on the experimental conditions. The corrosion rates for carbon steel A570 (*c1*, *c2*) coupons were higher (0.12, 0.13 mm year^−1^), compared to those for 1045 (*c3*, *c4*) coupons (0.08, 0.10 mm year^−1^). The lowest corrosion rates (0.04 mm year^−1^) were obtained for the abiotic controls. Therefore, the corrosion rates were about 2-3 times higher for coupons exposed to the water sample that contained bacteria compared to those of the abiotic controls. The existence of such differences could be due to the incubation conditions and their different chemical compositions. As mentioned in the Materials and Methods section, carbon steel A570 has a simpler composition compared to carbon steel 1045. Both aerobic and anaerobic bacteria could cause localized pitting or crevice corrosion on the carbon steel surface through the formation of biofilms [4,7,9,15]. These bacteria can coexist in biofilms, forming synergistic communities that influence the electrochemical processes through cooperative metabolic processes not observed in a single type of bacteria [8,11,13].

## 4. Conclusions

The aerobic and anaerobic bacteria from the water sample were able to initiate the corrosion of carbon steel A570 and 1045 coupons. The results of this study highlight that the carbon steel coupons exposed for 120 days to a water sample that contained bacteria revealed macroscopic and microscopic changes in the surface characteristics, and these changes could have been due to biofilm formation on their surfaces and the accumulation of the corrosion products. The corrosion rate varied from one type of carbon steel to another, depending on the incubation conditions and the chemical composition of the coupons. These results have important implications for a better understanding of the role of indigenous aerobic and anaerobic bacteria in the corrosion of carbon steel in aquatic environments.

## Figures and Tables

**Table 1:** Bacteria identification through phenotypic and genotypic characteristics.

Characteristics		Strain		
		IBB_Cn1_	IBB_Cn2_	IBB_Cn3_
Phenotypic	Color of colonies	creamy	red-yellow	white
	Gram	−	−	+
	Shape	rods	rods	rods
	Motility	+	+	+
	Respiratory type	FAn	FAn	An
	Catalase	+	+	+
	Oxidase	−	−	−
	Hydrogen sulfide production	+	−	+
	Pyocyanin pigment production	−	−	−
	Pyoverdin pigment production	−	−	−
	Growth on TTC medium	−	−	−
	Lactose utilization	+	+	−
Genotypic	RAPD using			
	AP5 primer (DFS, bp)	1000	−	−
	AP12 primer (DFS, bp)	300, 400, 650	300, 400, 650	250, 420
	PCR 16S rRNA gene using 27f-1492r primers (DFS, bp)	1465	1465	1465
	16S rRNA gene sequence, sequence identity (%)	*S. maltophilia*, 99.78%	*S. maltophilia*, 99.78%	*B. thuringiensis*, 99.85%
	GenBank accession number	MT893712	MT893713	MT893714

Facultative anaerobic (FAn) or anaerobic (An) growth on liquid LB medium; positive reaction (+); negative reaction (−); detected fragments size (DFS).

**Table 2:** Biocorrosion of carbon steel coupons exposed to the water sample.

CS Coupons	Bacteria Quantification (cells mL^−1^)						pH		Fe_2_O_3_ (wt.%)		CR (mm year^−1^)
	HB		IOB		SRB						
	0 Days	120 Days	0 Days	120 Days	0 Days	120 Days	0 Days	120 Days	0 Days	120 Days	120 Days
*c1*	4.0 × 10^1^	4.0 × 10^3^	4.0 × 10^0^	4.0 × 10^2^	3.5 × 10^0^	3.0 × 10^3^	7.70	5.60	5.13	90.42	0.12 ± 0.03
*c2*	4.0 × 10^1^	3.0 × 10^3^	4.0 × 10^0^	3.0 × 10^2^	3.5 × 10^0^	4.0 × 10^4^	7.70	5.80	5.13	86.97	0.13 ± 0.04
*c3*	4.0 × 10^1^	2.0 × 10^3^	4.0 × 10^0^	4.0 × 10^2^	3.5 × 10^0^	4.0 × 10^5^	7.70	6.00	5.13	79.46	0.08 ± 0.01
*c4*	4.0 × 10^1^	4.0 × 10^2^	4.0 × 10^0^	3.0 × 10^2^	3.5 × 10^0^	5.0 × 10^5^	7.70	6.02	5.13	90.19	0.10 ± 0.02
*Ac*	−	−	−	−	−	−	7.70	7.70	5.13	38.50	0.04 ± 0.01

Carbon steel (CS) A570 (*c1*, *c2*), 1045 (*c3*, *c4*) coupons immersed in the water sample and incubated (0–120 days) under aerobic (*c1*, *c3*) or anaerobic (*c2*, *c4*) conditions; abiotic controls (*Ac*); most probable number per mL (cells mL^−1^), heterotrophic bacteria (HB), iron-oxidizing bacteria (IOB), sulfate-reducing bacteria (SRB); the values represent the average from two independent assays; pH evaluation; Fe_2_O_3_ quantification (wt.%); corrosion rate (CR) of CS coupons with standard deviation (mm year^−1^).

## Data Availability

Not applicable.
